# Molecular diagnostics in the evaluation of thyroid nodules: Current use and prospective opportunities

**DOI:** 10.3389/fendo.2023.1101410

**Published:** 2023-02-24

**Authors:** Jena Patel, Joshua Klopper, Elizabeth E. Cottrill

**Affiliations:** ^1^ Department of Otolaryngology – Head & Neck Surgery, Thomas Jefferson University Hospital, Sidney Kimmel Medical College, Philadelphia, PA, United States; ^2^ Department of Medical Affairs, Veracyte, San Francisco, CA, United States

**Keywords:** thyroid nodule, thyroid cancer (TC), diagnosis, prognosis, targeted therapy, molecular markers

## Abstract

Thyroid cancer is the most common endocrine malignancy with an estimated 43,800 new cases to be diagnosed in 2022 and representing the 7th most common cancer in women. While thyroid nodules are very common, being identified in over 60% of randomly selected adults, only 5-15% of thyroid nodules harbor thyroid malignancy. Therefore, it is incumbent upon physicians to detect and treat thyroid malignancies as is clinically appropriate and avoid unnecessary invasive procedures in patients with benign asymptomatic lesions. Over the last 15-20 years, rapid advances have been made in cytomolecular testing to aid in thyroid nodule management. Initially, indeterminate thyroid nodules, those with Bethesda III or IV cytology and approximately a 10-40% risk of malignancy, were studied to assess benignity or malignancy. More recently, next generation sequencing and micro-RNA technology platforms have refined the diagnostic capacity of thyroid nodule molecular testing and have introduced opportunities to glean prognostic information from both cytologically indeterminate and malignant thyroid nodules. Therefore, clinicians can move beyond determination of malignancy, and utilize contemporary molecular information to aid in decisions such as extent of surgery and post-therapy monitoring plans. Future opportunities include molecularly derived information about tumor behavior, neo-adjuvant treatment opportunities and response to thyroid cancer therapies.

## Introduction

Thyroid cancer is the most common endocrine malignancy with an estimated 43,800 expected new cases diagnosed in 2022 and representing the 7^th^ most common cancer in women (1). Thyroid cancer almost always presents as a thyroid nodule and thyroid nodules are very common with over 60% of the population having one or more by the time patients reach their 7^th^ and 8^th^ decades of life (2). However, only 5-15% of thyroid nodules harbor thyroid malignancy. Fine needle aspiration cytology (FNAC) is the foundation for diagnosis of nodules that meet criteria for biopsy, and a Bethesda II (BII - benign) or Bethesda VI (BVI - malignant) cytology result has excellent accuracy and correlation with final histopathology upon surgical resection (2–4). BII cytology predicts benign histology 97% of the time or greater and BVI cytology confers a risk of malignancy up to 99% (4). The primary challenge in the evaluation of thyroid nodules occurs in the setting of Bethesda III (BIII) or Bethesda IV (BIV) cytology, often grouped together as indeterminate thyroid nodules (ITN).

Approximately 20-25% of thyroid nodule aspirates result in ITN cytology (5). The risk of malignancy of BIII and BIV ITN ranges from 6-40% depending on the institution and the categorization of noninvasive follicular thyroid neoplasm with papillary-like nuclear features (NIFTP) as benign or malignant (4). Historically, consensus guidelines recommended surgery, often in the form of a thyroid lobectomy, for definitive diagnosis of ITN since it is often not possible to differentiate between benign and malignant nodules by cytology alone (6, 7). This approach is sub-optimal given the cost, possible morbidity, and need for thyroid hormone replacement in a subset of patients after lobectomy and all patients after total thyroidectomy; especially since ~75% of ITN will prove to be benign on final histopathology (4, 8, 9). The utilization of transcriptional signatures and discovery of driver mutations promoting thyroid cancer development and influencing its behavior provided the molecular foundation for improved diagnostic accuracy in ITN (10, 11). As will be described, molecular diagnostics has moved beyond aiding in diagnosis and can provide information on tumor prognosis (12).

The goal of this review is to provide an update on commercially available lab developed molecular diagnostic tests for use in nodular thyroid disease. The contemporary clinical use, advantages, and disadvantages, as well as future potential applications will be discussed.

## Diagnostic test performance metrics review

**Figure 1:**
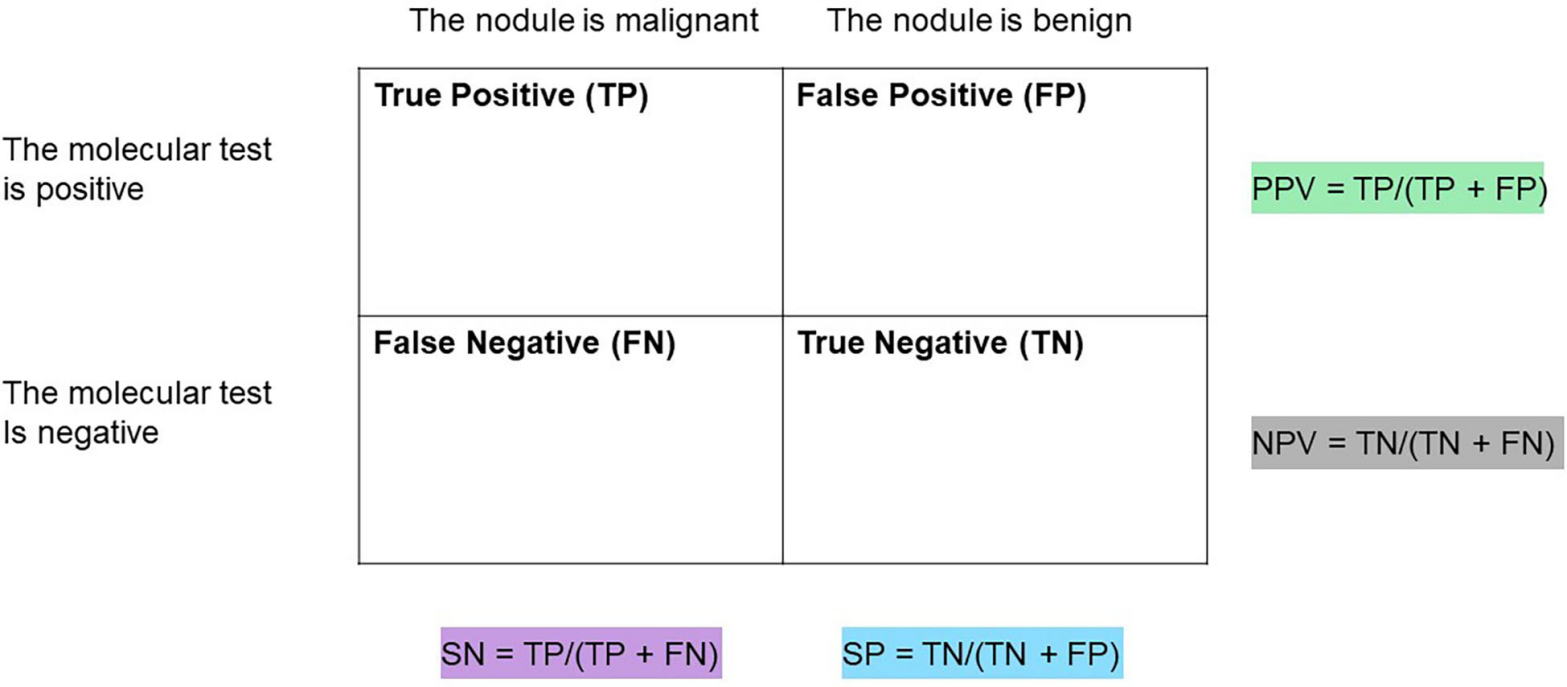
Table to assist in calculations of sensitivity (SN), Specificity (SP), positive predictive value (PPV), and negative predictive value (NPV) of a diagnostic test.

A brief review of test sensitivity (SN), specificity (SP), negative predictive value (NPV), and positive predictive value (PPV) is warranted to promote appropriate understanding and scrutiny of molecular diagnostic performance metrics (**Figure 1**) (13–15). SN is a calculation of the number of true positives (for this topic, the patient has thyroid cancer, and the molecular test reports a positive finding) divided by all the patients with thyroid cancer (who have true positive plus false negative test results). A low SN indicates thyroid cancers have been missed (called negative or benign) by the molecular marker test. Alternatively, SP is a calculation of the true negatives (the patient does not have thyroid cancer and the test is negative) divided by all the patients without thyroid cancer (true negative plus false positive test results) (**Figure 1**) (15).

**Figure 2:**
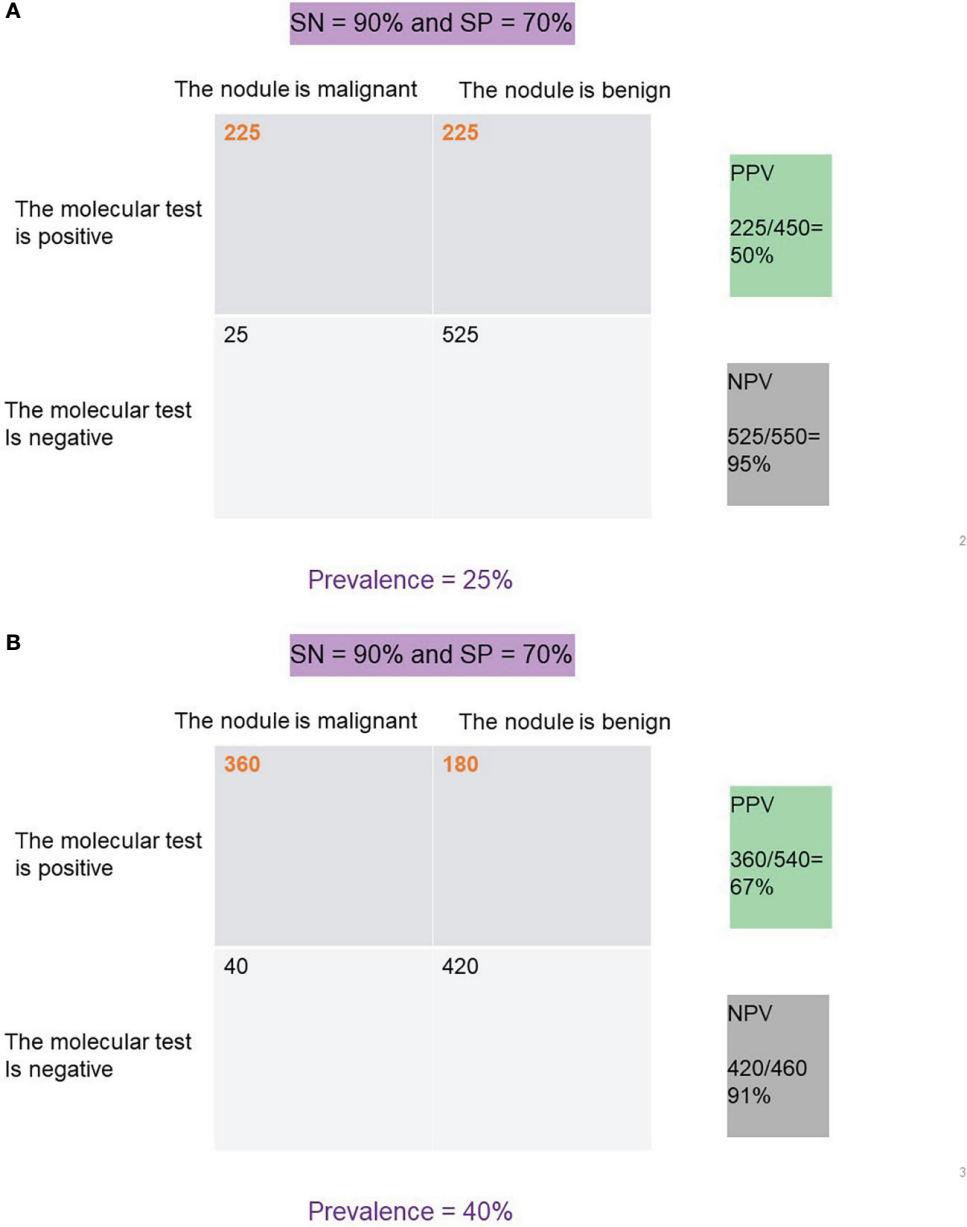
**(A)** PPV and NPV for a test with 90% SN and 70% SP at a disease prevalence of 25%. **(B)** PPV and NPV for a test with 90% SN and 70% SP at a disease prevalence of 40%.

Clinically, NPV and PPV are better indicators of a test’s ability to rule out or rule in disease, respectively. NPV is a calculation of the true negatives divided by all the patients with a negative test result (true negatives and false negatives). PPV is a calculation of the true positives divided by all the patient with a positive test result (true positives and false positives) (13, 14). At any given SN and SP, both NPV and PPV are affected by the disease prevalence in the population such that a higher disease prevalence will result in a higher PPV and lower NPV than in a population with a lower prevalence of disease (**Figure 2**).

Other measures of diagnostic performance include overall accuracy, which is the proportion of correctly identified patients (true positive and true negative results) relative to the entire cohort, and likelihood ratios, the probability of the expected test result in those with thyroid cancer as compared to the same result in those without (16).

It is critical that a thyroid nodule molecular diagnostic test is validated with a high-quality study that ideally is prospective, multi-center, with blinded central histopathologic review. A prospective validation study reduces clinical decision-making bias regarding who enters the study cohort and who has surgery. A multi-center study with blinded histopathology review confirms the gold standard presence or absence of disease in a broad and representative population which aids in reliable SN and SP calculations. Finally, all patients enrolled in the study must have surgery so the prevalence of thyroid cancer in the studied cohort can be known and utilized to calculate the NPV and PPV.

## A brief history of molecular diagnostic laboratory assays

The utilization of molecular diagnostics has rapidly advanced over the last 10-15 years with some older generation tests maintaining a presence for use and others being replaced by next generation sequencing (NGS) platforms. A brief review of older and currently unavailable molecular tests is presented, primarily to provide context for assessing the currently available tests.

The identification of the *BRAF*V600E mutation in papillary thyroid carcinoma (PTC) in 2003 was one of the earliest identified molecular signatures correlating a molecular variant with final histology (17). *BRAF*V600E is a highly specific yet poorly sensitive marker for thyroid cancer, especially in ITN where it is now known that *BRAF*V600E is present in <10% of molecularly tested ITN aspirates (18). Thus, research began into mutation panels that raise test SN to detect more malignant nodules. One of the first studies was a prospective multi-institutional study evaluating *BRAF*V600E, *BRAF*K601E, mutations of *NRAS*, *KRAS* and *HRAS* gene codons as well as *RET/PTC 1/3* rearrangements and *PAX/PPARγ* fusions (19). This panel showed a high specificity with 97% of mutation positive nodules representing histologically malignant tumors yet only a 62% sensitivity as not all malignancies carried variants or fusions detected by the panel.

In 2012, the clinical validation study of the Afirma^®^ Gene Expression Classifier (GEC) was published. The Afirma GEC combined mRNA expression on a 167-gene microarray platform with machine learning with a goal of predicting benign nodules with ITN cytology to reliably rule out thyroid cancer and avoid unnecessary surgery (10). This was a prospective, multi-center study with blinded central histopathology review and reported a high sensitivity of 90% and a high negative predictive value of 94% [(95% confidence interval (CI)), 87-98)] across BIII and BIV nodules) By virtue of the test design with an emphasis on ruling out thyroid cancer, the specificity and PPV were relatively low. As the first rule-out test, there was caution regarding the possibility of false-negative results among potentially more aggressive cancers. Knowing that the standard treatment of ITN nodules was surgery, a Hurthle cell cassette was included with the GEC to intentionally call most Hurthle samples as GEC suspicious. Resultingly, the overall specificity of the GEC amongst Hurthle cell lesions was only 12% (10, 20). The acceptance and comfort with rule-out testing amongst physicians, the need for a higher benign call rate and PPV, combined with scientific advances and reduced costs of next-generation sequencing prompted development of the Afirma Genomic Sequencing Classifier (GSC) (21).

Thyroseq^®^ has evolved with multiple iterations expanding the number of molecular variants identified from the initial 7 gene panel (targeted variants in 4 genes and 3 gene fusions) to a targeted NGS platform including 12 genes in version 1 to 14 genes analyzed for point mutations and 42 types of gene fusions in version 2 (22, 23). Thyroseq v2 data was published in 2013 and the expanded Thyroseq v2.1 panel data was published in 2015. Thyroseq v2.1 reported test performance was a sensitivity of 90.9% [CI 78.8–100], specificity of 92.1% [CI 86.0–98.2], positive predictive value of 76.9% [CI, 60.7–93.1], and negative predictive value of 97.2% [CI 78.8–100], with an overall accuracy of 91.8% [CI, 86.4–97.3] These earlier versions of Thyroseq NGS panels were not tested in prospective, multi-center studies with blinded histopathologic review (23). As will be described, Thyroseq v3, the current commercially available testing platform, further expanded the number of molecular variants and fusions tested.

Other molecular tests that were used for the preoperative diagnosis of ITN included a combined miRNA and somatic gene mutation panel from Asuragen^®^ (available ~2010-2014) and the micro-RNA (miRNA) classifier RosettaGX^®^ Reveal (available from ~2016-2018) (24, 25). Neither is currently commercially available.

## Utilization of molecular diagnostics in clinical practice

The incorporation of thyroid nodule molecular diagnostic testing into clinical practice bears some discussion. Thyroid nodule biopsies can occur in outpatient clinics, pathology departments, radiology suites, and rarely in an inpatient setting. Each practice, institution, and location present opportunities and challenges.

One consideration is whether to utilize a “collect on all” protocol where a sample for molecular marker testing is collected at the time of a thyroid nodule’s initial FNA. Alternatively, patients can be asked to return for a repeat FNA for collection of a sample for molecular testing after an indeterminate cytology result. Given most biopsies are read as definitively benign or malignant (approximately 75%), allowing a patient to avoid unnecessary needle passes is reasonable. However, the inconvenience of taking more time from work or away from home, additional copays, and a repeat of the FNA preparation and procedure argues for collecting a molecular marker sample at the time of the initial FNA in the event of an ITN result. Most patients will be in favor of getting all samples collected at once in lieu of returning for a second procedure if given the option. Collecting on all samples does require tracking of specimens, a timely send out of material upon receipt of an ITN result and discarding of unused samples to free up space for future samples. This does require dedicated organization and effort. Currently, the Afirma and Thyroseq testing platforms both allow for centralized cytology diagnosis (at Thyroid Cytology Partners and CBL Path respectively) with reflex send out of collected molecular samples upon an ITN result. ThyGeNEXT/ThyraMIR^®^ (MPTX) offers cytology reads *via* a partnership with Dianon Pathology. In a community practice setting, a transition from decentralized thyroid FNAs in radiology practices, with separate cytology reads at individual centers, to a centralized collection for cytology and molecular markers resulted in a decrease of ITN from 24% to 10% and a reduction in diagnostic surgeries from 24% to 6% (26).

If onsite cytology assessment is available, this may represent the best model. At the time of the FNA, rapid on-site evaluation can be made to determine cytology adequacy, diagnosis, and the need for extra needle passes for dedicated material for molecular testing while a patient is prepped and waits. This practice can reduce nondiagnostic aspirates and improve diagnostic accuracy (27). The logistics of this practice demand an integrated clinic model with enough pathology personnel to create cytology slides and have a rapid read. This is not feasible in many, if not most, clinical settings.

Slide scraping, the collection of thyroid follicular cells from cytology slides with the aid of microscope assisted microdissection, presents a convenient methodology for running some molecular tests on cytology smears when the patient has not had access to molecular diagnostics or there was no collection of a molecular sample at the time of initial FNAC. The Afirma platform does not offer slide scraping while MPTX and Thyroseq do offer this collection method. Though convenient, there are limitations to slide scraping relative to collecting a fresh sample. In the MPTX validation study, 18% of slides failed to provide adequate nucleic acid quantity to run the assay (28). In the Thyroseq validation of slide scraping, Diff-Quik stained smears were inadequate 35% of the time though all Papanicolaou-stained smears were informative (29). Of greater concern than assay failure, are the discordant results between microdissected cytology smears relative to a fresh FNAC placed in its respective nucleic acid protection/storage buffer. There was 11% discordance for miRNA with the ThyraMir portion of MPTX and 14% of copy number alterations along with 17% of fusions were missed (false negatives) on Thyroseq slide scraping compared to a fresh sample from FNAC (29, 30). Clinicians should consider the discussion point regarding the use of slide scraping for Thyroseq, “the collection of a portion of a fresh FNA sample directly into a nucleic acid preservative solution should be attempted whenever possible because this provides the highest success rate and accuracy of testing” (29).

## The role of molecular diagnostics in ITN for benign vs malignant diagnosis

Molecular testing has become a more commonly utilized tool in the clinical setting to help provide additional risk information for ITN. Ideally, the results of the molecular test shift the risk of malignancy (ROM) from ~25% with ITN cytology to risks that help determine which patients will benefit from conservative surveillance versus definitive surgical intervention (31, 32). Molecular testing platforms have evolved with technical advancements coming in the form of expanded genomic information and improved test performance. As of this writing, the three most used molecular tests in the United States include the Afirma Genomic Sequencing Classifier (Afirma GSC), ThyGeNEXT/ThyraMIR (MPTX), and Thyroseqv3 (TSv3). Each molecular test is performed using a different method; however, all three aim to provide the clinician with accurate and precise information concerning patients’ risk of nodule malignancy. To our knowledge there is no widespread use of these molecular markers outside of the United States. There is limited use in certain provinces of Canada as well as sporadic use in South America and Europe, almost universally without national healthcare or insurance support.

The Afirma GSC uses next generation RNAseq and whole transcriptome analysis combined with machine learning algorithms to provide a benign or suspicious result in nodules with ITN (21). MPTX is a multiplatform test approach that combines a next generation targeted sequencing panel (ThyGeNEXT) with a microRNA risk classifier test (ThyraMIR) (28). TSv3 is a targeted next generation sequencing test that evaluates point mutations, gene fusions, copy number alterations and abnormal gene expression in 112 thyroid cancer related genes. A high-quality diagnostic test validation study that is prospective, blinded, multi-center and representative of the intended test population is critical to provide confidence in the test performance. Post-validation real-world studies are important for increasing confidence in a test’s performance and providing evidence of benefit in clinical practice outside of the controls of a validation study.

MPTX screens samples with the ThyGeNEXT NGS panel that include selected DNA mutations in the following genes: *ALK, BRAF, GNAS, HRAS, KRAS, NRAS, PIK3CA, PTEN, RET* and *TERT* promoter genes. The following gene fusions are detected by analysis of RNA: *ALK*, *BRAF, NTRK, PPARγ, RET* and *THADA* (28). If there is a strong driver mutation detected, the sample is considered positive. If the sample has a weak oncogenic driver mutation or no mutation, it is further risk stratified using the microRNA classifier (ThyraMir). The initial ThyraMir panel included 5 growth-promoting miRNAs (miR-31, -146, -222, -375, -551) and 5 growth-suppressing miRNAs (miR-29, -138, -139, -155, -204). MPTX results are ultimately reported as one of three categories (negative, moderate, or positive) based on results of the combined ThyGeNEXT mutation panel and ThyraMIR microRNA risk classifier thresholds (28).

The MPTX has been analytically validated and the clinical validation study is a retrospective, blinded multicenter study (28, 33) (**Table 1**). Unanimous histopathology consensus was not met in 19% of cases which were excluded from analysis. MPTX results for 197 subjects with ITN were categorized as positive, moderate risk or negative for malignancy from a cohort with a 30% disease prevalence. Moderate risk was assigned to 28% of the cohort who are estimated to have the same ROM as the baseline cancer prevalence of 30%. When the moderate risk patients were found to have malignant histology, they were assigned as true positives. When the moderate risk patients were found to have benign histology, they were assigned as true negatives. Thus, the moderate risk groups were categorized in a way that bolsters overall test SN and SP (more true positives or true negatives than defined by the positive or negative groups alone). However, the moderate risk subjects/results were not used in the PPV and NPV calculations. Finally, based on concerns that the proportion of histologic subtypes within the studied cohort were inconsistent with published literature, a prevalence adjustment calculation was made to match the reported proportions of adenomas, malignant subtypes and NIFTP as reported by the TSv3 validation study (28, 34). Bearing these considerations, the results showed 95% SN [CI, 86- 99] and 90% SP [CI, 84-95] for disease. Negative MPTX results ruled out disease with 97% NPV while positive MPTX results ruled in high-risk disease with a 75% PPV. An updated ThyGeNEXT panel improved strong driver mutation detection by 8% with *BRAF*V600E and *TER*T promoters being the most common mutations. Additionally, this newer panel increasingly detected coexisting drivers by 4%, TERT being the most common and often paired with *RAS* (35). A pairwise analysis of miRNA to detect medullary thyroid cancer (MTC) showed 100% accuracy on a study of 4 MTC and 26 non-MTC samples (36). Finally, MPTX has recently been updated with the addition of miR-21 and an interdependent pairwise microRNA expression analysis (MPTXv2). This updated MPTX platform was tested on the same cohort as the original validation study population. The results showed a decrease in the moderate-risk cohort from 28% to 13% (p < 0.001) and a reported improvement in PPV to 96% (from 74%) and NPV to 99% (from 95%) (p=NS for both) (37).There have been no completely independent research studies to assess the MPTX performance. In one analysis of pediatric lesions comprised of 66 malignant and 47 benign tumors, MPTXv1, analysis performed with 70% SN and 96% SP (38).

**Table 1:** Validation study summary of the most used thyroid nodule molecular diagnostic tests in the United States.

	Afirma GSC	Thyroseq v3	ThyGeNEXT/ThyraMIR
Test Type	Whole transcriptome RNA NGS	Targeted DNA and RNA NGS	Targeted NGS + miRNA expression
Validation Study Study Design	Patel et al (20) Prospective	Steward et al (33) Prospective	Lupo et al (27) Retrospective
Sample Size	190	286	178
Specificity	68%	82%	90%
Sensitivity	91%	94%	93%
NPV	96%	97%	95%
PPV	47%	66%	74%

The Afirma GSC samples are initially tested for RNA quantity and quality. Sufficient samples are tested against initial classifiers to detect parathyroid tissue, MTC, *BRAF*V600E variants and *RET/PTC*1 and *RET/PTC*3 fusions. Recently, the validation of the MTC classifier of the Afirma GSC showed 100% SN and 100% SP in a cohort of 21 MTC and 190 non-MTC lesions (39). If all the classifiers are negative and there is adequate follicular content, the GSC ensemble model relies heavily on differential gene expression of > 10,000 genes for sample classification of GSC-B or GSC-S results. The Afirma GSC clinical validation study was based on a cohort of ITN samples collected prospectively from multiple community and academic centers from the Afirma GEC validation (10). All patients underwent surgery without known genomic information and all samples were assigned a histopathology diagnosis by an expert panel blinded to all genomic information. The results showed (at a 24% cancer prevalence): SN - 91% [CI, 79-98], SP - 68% [CI, 60-76], NPV - 96% [CI, 90-99], PPV - 47% [CI, 36-58] (21) (**Table 1**). Since the validation study, 14 independent real-world studies have been published and in aggregate show a significant improvement in performance over the Afirma GEC, primarily with improved specificity and a higher benign call rate (BCR) of 65% (as compared to 54% with the Afirma GEC) (40–54). As expected, some of these studies have also demonstrated that the implementation of Afirma GSC reduced the rate of surgical intervention by 45-68% (40, 43). A meta-analysis by Vuong et al. including seven studies comparing the performance of Afirma GEC to GSC and found that GSC had a higher BCR (65.3% vs 43.8%; P <0.001), a lower resection rate (26.8% vs 50.1%; P <0.001), and a higher risk of malignancy (60.1% vs 37.6%; P <0.001) in resected specimens (55) (**Table 2**).

The Afirma GSC incorporates Hurthle/oncocytic and neoplasm classifiers to enhance the diagnostic accuracy in predominately oncocytic ITN relative to the Afirma GEC (20). A review of four independent post validation studies of the Afirma GSC performance in oncocytic cell lesions showed maintenance of a high SN (3 with 100% SN and one with 80% SN) and improved SP (81-100% for GSC compared to 29-43% for GEC) (56). When compared to the GEC, the BCR for oncocytic cell–predominant nodules by the GSC is significantly elevated (73.7% vs 21.4%; P < 0.001) (55).

TSv3 is a genomic classifier (GC) where a value is assigned to each detected genetic alteration based on the strength of association with malignancy: 0 (no association), 1 (low cancer risk), or 2 (high cancer risk). A GC score calculated for each sample is a sum of individual values of all detected alterations, with GC scores 0 and 1 accepted as test negative (score 1 is commercially reported as “currently negative”) and scores 2 and above as test positive (57). “Currently negative”, low cancer probability alterations, are included in the BCR in TSv3 studies. The clinical validation study for TSv3 by Steward et al. was a prospective, multi-center, blinded study that ultimately analyzed 257 ITN, all with histologic consensus. The test demonstrated a 94% [CI, 86%-98%] SN and 82% [CI, 75%-87%] SP. With a cancer/NIFTP prevalence of 28%, the NPV was 97% [CI, 93%-99%] and PPV was 66% [CI, 56%-75%] (34) (**Table 1**). There have been 10 independent studies assessing the performance of TSv3 (46, 47, 58–65). A recent meta-analysis by Lee at al. including six studies (total 530 thyroid nodules) evaluating the performance of TSv3 found a similar sensitivity of 95.1% [CI, 91.1–97.4%] but a lower specificity of 49.6% [CI, 29.3–70.1%] when compared to the original validation study; the reported PPV of 70% [CI, 55–83%], and NPV of 92% [CI, 86–97%] remained comparable (66) (**Table 2**).

**Table 2:** Meta-analysis data of Afirma GSC and Thyroseq v3.

	Afirma GSC	Afirma GSC	Thyroseq v3
Meta-analysis	Vuong et al (52)	Lee et al (53)	Lee et al (53)
# Included Studies	7 studies	7 studies	6 studies
Sample Size	807	472	530
Specificity	43%	53%	50%
Sensitivity	94.3%	96%	95%
NPV	90%	96%	92%
PPV	63.1%	63%	70%

Molecular tests can be classified as “rule in” vs “rule out” based on their ability to confirm or exclude malignancy. Vargas-Salas et al. found that with a thyroid cancer prevalence of 20–40%, a robust “rule-out” test requires a minimum NPV of 94% and a minimum sensitivity of 90%, whereas to “rule- in” malignancy, a test requires a PPV of at least 60% and a specificity above 80% (67). MPTX, Afirma GSC, and TSv3 all perform well as “rule out” tests for ITN based on their relatively high sensitivities and NPVs, though independent confirmation of MPTX performance is lacking. MPTX has too few studies to compare its performance to other molecular testing platforms and future studies are needed to confirm its clinical efficacy. A study by Silaghi et al. comparing the performance of Afirma GSC and TSv3 found TSv3 to have the best overall diagnostic performance with the lowest negative likelihood ratio (NLR 0.02), followed by Afirma GSC (NLR 0.11). Both TSv3 and Afirma GSC achieved optimal results to exclude malignancy; however, both failed to achieve a higher performance to confirm or “rule in” a malignancy when compared to their predecessor Thyroseqv2 (68). Similarly, Lee et al. found there was no statistically significant difference in diagnostic performances between the Afirma GSC and TSv3 (66) (**Table 2**). Finally, Livhits et al. performed a randomized clinical trial by using Afirma GSC or TSv3 in routine clinical practice on a rotating monthly basis. They found that both Afirma GSC and TSv3 have a relatively similar specificity (80% and 85%, respectively), and both allowed approximately 49% of patients with indeterminate nodules to avoid diagnostic surgery (46). Given the similar performance, it is no longer accurate to call Afirma a “rule out test” and Thyroseq a “rule in test” as they have been commonly described with earlier iterations of the testing platforms and in a recent review (69).

## The role of molecular genetic testing in predicting thyroid cancer prognosis

Molecular genetic testing is a valuable tool in understanding patients’ prognosis based on specific mutations detected in thyroid cancer. Various mutations are associated with increased tumor aggressiveness, metastatic lymph node spread, a tendency to de-differentiate, and/or reduced efficiency of radioiodine treatment. The main known genetic causes of thyroid cancer include point mutations in the *BRAF, RAS, TERT* promoter*, RET*, and *TP53* genes and the fusion genes *RET/PTC*, *PAX8/PPARγ*, and *NTRK* (70). Molecular genetic testing of thyroid tissue in the preoperative and/or postoperative period is becoming more common, and therefore detection of genetic changes may serve as a prognostic factor that can help determine the extent of surgical treatment and the use of systemic targeted therapy. The characterization of molecular variants and fusions as *BRAF*-like, *RAS*-like, and non-*BRAF* non-*RAS*-like has helped to group molecular alterations in thyroid cancer that share similar risk of events such as extra-thyroidal extension and lymph node metastases (71, 72). For example, a retrospective analysis by Tang et al. associating pathologic features to the aforementioned molecular classes showed a statistically higher rate of T4 tumor size and N1b nodal metastases in *BRAF*-like mutated tumors (22%) compared to the other classes (≤ 6%) amongst other more aggressive findings (12). Afirma GSC, MPTX, and TSv3, have shown promise in predicting disease recurrence in thyroid cancers and Bethesda V/VI nodules based on the detection of low-risk vs high-risk genetic mutations.

In thyroid nodules with Afirma GSC suspicious results, or thyroid nodules with BV or BVI cytology, Afirma Xpression Atlas (XA) can provide more granular molecular information. The analytical and clinical validation of XA, which identifies thyroid nodule molecular variants and fusions by whole transcriptome sequencing, was published in 2019 (73). In 2020, the panel was expanded to detect molecular alterations in 593 genes allowing XA to report on 905 variants and 235 fusions. Afirma XA results may offer important prognostic insights; for example, nodules with a non-*RAS* and non- *BRAF* molecular profile have lower rates of lymph node metastasis and extrathyroidal extension (74). A large retrospective study by Hu et al. demonstrated that 44% of Bethesda III/IV Afirma GSC-S and most Bethesda V/VI nodules (87% BVI) had at least one genomic variant or fusion identified, which could optimize individual treatment decisions (18). The ability of Afirma XA to demonstrate improved clinical outcomes based on surgery and mutational status is yet to be determined as no randomized trials have been performed; however, the genomic insights provided by XA may predict tumor aggressiveness and provide important information regarding variants for targeted therapy (75).

Labourier et al. found that in a systematic review of the literature, 70%-75% of malignant/Bethesda VI cytology were expected to be positive for the oncogenic *BRAF*V600E substitution with the second most frequent gene alteration being *TERT* promoter mutations (11%) (76). High frequency of oncogenic *BRAF* mutations has important clinical implications and multiple studies have shown that *BRAF*V600E correlates with aggressive features of thyroid cancer such as extrathyroidal extensions, vascular invasion, larger thyroid nodule size, advanced staging, lymph node metastasis and recurrence (77). Additionally, *TERT* promoter mutations are among the most recognized markers associated with aggressive thyroid cancer phenotypes (77).

When specifically evaluating the performance of TSv3 in thyroid nodules with Bethesda V (suspicious for malignancy) cytology, Skaugen et al. found that TSv3 had sensitivity of 89.6% (95% CI, 82.4%- 94.1%) and specificity of 77.3% (95% CI, 56.6%-89.9%). Moreover, when TSv3 positive Bethesda V nodules were sorted into molecular risk groups (low, intermediate, high), disease recurrence was more commonly found in the high-risk group whereas no patients in the low-risk group developed recurrence (78). Another study by Hescot et al. used TSv3 to determine if there were molecular prognostic factors associated with recurrence and overall survival in patients’ with poorly differentiated thyroid carcinomas (PDTCs). Of the 40 patients tested with TSv3, high-risk molecular signatures (*TERT*, *TP53* mutations) were found in 24 cases (60%), intermediate-risk signature in 9 cases (22.5%) and low-risk signature in 7 cases (17.5%) with potentially actionable mutations that may be amenable to targeted therapy identified in 10% of cases. Furthermore, the high molecular-risk signature was associated with distant disease metastasis (P = 0.007) and with worse overall survival (P = 0.01), whereas none of the patients with low-risk molecular signature died due to thyroid cancer (79).

It is important to note that there are no established guidelines addressing management decisions based on the detection of most genetic alterations detected in thyroid nodules regardless of cytology category. In ITN, the most studied value is in the diagnosis of benignity or malignancy. The value of knowing the molecular alterations in BV and BVI thyroid nodules has yet to be investigated in prospective multi-center studies. Additionally, molecular tests performance metrics are generally assessed independent of other clinically relevant factors such as family history of thyroid cancer, heritable syndromes, radiation exposure, and thyroid ultrasound features. One area of increasing interest is the identification of aggressive thyroid cancers that may be amenable to future systemic targeted therapies as needed, possibly in the neo-adjuvant setting.

## Molecular identification of targetable alterations in thyroid cancer

While the use of molecular testing to risk-stratify indeterminate thyroid nodules is encouraging, arguably the most exciting use of this technology is in the setting of advanced and aggressive thyroid disease where identification of targetable mutations can have significant clinical impact (**Table 3**). In differentiated thyroid cancer, the overall mortality is low, however 15% of cases will be locally invasive and in those with distant metastases which are radioioine (RAI)-refractory, the 10-year overall survival is <50% (86, 87). Conversely, the most aggressive subtypes of thyroid cancer, medullary, poorly differentiated, and anaplastic, have high disease-specific mortality. Especially in these thyroid cancer subsets with high mortality rates, there has been substantial expansion of the therapeutic armamentarium with tumor genome-directed therapies over the past decade (80, 88–90). Studies have identified several targetable (or potentially targetable) alterations in advanced thyroid cancer, including mutations in commonly detected genes such *BRAF*V600E, *RET*, *PIK3CA*, as well as gene fusions including *RET*, *NTRK*, and *ALK*. In addition to therapies targeting specific genetic alterations, immunotherapy shows significant promise in treating tumors with microsatellite instability, high tumor mutational burden (TMB), and high PD-L 1 expression. With the possibility of identifying genomic alterations *via* NGS in advanced thyroid cancers, the study of neoadjuvant therapy for aggressive disease has just begun.

**Table 3:** FDA approved molecularly targeted therapies in thyroid cancer.

Dabrafenib (80) (BRAF inhibitor) & Trametinib (80) (MEK inhibitor)	Larotrectinib (81, 82) (Selective TRK inhibitor)	Entrectinib (83) (multi-kinase inhibitor NTRK1/2/3, ROS1, & ALK)	Selpercatinib (84) (Selective RET kinase inhibitor)	Pralsetinib (85) (Tyrosine Kinase Inhibitor)
• 18 years old • locally advanced, unresectable or metastatic solid tumors • ***BRAFV600E mutant-positive***	• 1 month old • locally advanced or metastatic solid tumors • Tumor agnostic • ***NTRK fusion-positive***	• 18 years old • locally advanced or metastatic solid tumors • penetrate blood-brain barrier • Tumor agnostic • ***NTRK fusion-positive***	• 12 years old • *RET*-driven advanced or metastatic cancer • ***RET mutant-positive*** Medullary Thyroid Cancer • ***RET fusion-positive*** radioactive iodine-refractory thyroid cancers	• 12 years old • *RET*-driven advanced or metastatic cancer • ***RET mutant-positive*** Medullary Thyroid Cancer • ***RET fusion-positive*** radioactive iodine-refractory thyroid cancers

Recently, a multidisciplinary, multi-institutional, multi-national consensus statement was jointly published by the American Head and Neck Society (AHNS) and the International Thyroid Oncology Group (ITOG) defining advanced thyroid cancer and its targeted treatment (91). The group advocates for molecular testing to be “performed in Clinical Laboratory Improvement Amendments (CLIA)- accredited laboratories (or their international equivalent), on appropriate specimens, using clinically validated procedures, which may include laboratory-developed tests or FDA-approved commercial assays” (82). With the support of high-quality evidence, the consensus recommends that “when somatic mutational testing is performed for thyroid cancer, multiplexed NGS-based panels are superior to multiple single-gene tests” and that, “NGS panels that include assays for gene fusions are preferred given the ability to detect multiple mutations and fusions in one assay thereby conserving tissue and limiting expense” (80).

Differentiated Thyroid Cancer (DTC): Accounting for roughly 95% of thyroid cancers, DTC arises from follicular thyroid cells and is often RAI-avid. This allows the vast majority of DTC to be treated with surgery alone for smaller tumors or surgery with RAI and levothyroxine suppression therapy for more advanced or aggressive disease. However, it is reported that 7–23% of patients with DTC will develop distant metastases, and two-thirds of patients with distant metastases become RAI-refractory (86, 90). These patients have poor prognosis with overall 10-year survival of <50% (86, 87). Multicenter, randomized, double-blind, placebo-controlled, phase III studies led to FDA approval of multi-kinase inhibitors (MKIs) Sorafenib and Lenvatinib, for the treatment of RAI-refractory locally advanced (non-operative) or metastatic DTC (80, 90). MKIs block activation of several key receptors that regulate thyroid cancer progression including angiogenesis. While studies showed progression free survival (PFS) benefit in the treatment groups compared to placebo groups (80, 90), because of the non-specific targeting of these drugs, their clinical utility is limited by their substantial toxicity profiles.

In the last decade, recognition of important molecular drivers and signaling pathways has led to the development of molecular-targeted therapies especially for advanced and RAI-refractory differentiated thyroid cancer. Presence of a *BRAF* V600E mutation, the most common driver mutation in the spectrum of follicular cell derived thyroid cancers, can confer susceptibility to selective RAF kinase inhibitors in some cancer lineages. The combination of dabrafenib (*BRAF* inhibitor) and trametinib (*MEK* inhibitor), which was initially FDA-approved in *BRAF*V600E mutated ATC, has also been studied in *BRAF*-mutated PTC with high response rates (50% single-agent dabrafenib vs. 54% combination, modified RECIST criteria) and median progression free survival 11.4 vs. 15.1 months. This combination of drugs recently garnered approval for treatment of *BRAF*-mutated DTC (83). The FDA-approved drugs selpercatinib and pralsetinib target the oncogenic *RET* gene fusions, detected in approximately 10% of PTC (81, 92). Thyroid cancers harboring genetic rearrangements involving *NTRK*1/3 (~2% of PTC) can respond to treatment with TRK inhibitors, including FDA-approved larotrectinib and entrectinib (72, 93–95). *ALK* fusions are still more rare in well differentiated thyroid cancers (<1% of PTC) but are identified more frequently in PDTC. *ALK*-inhibitors are FDA-approved for solid tumors that harbor *ALK* fusions and a few patients with thyroid cancer have been included in the reported clinical trials and/or case reports, although no ALK-inhibitors are currently FDA-approved for DTC specifically. Therefore, *ALK* fusion testing is currently indicated for advanced DTC only in the context of either “off-label” treatment or clinical trials. Lastly, while microsatellite instability (MSI) and TMB in DTC are often low, MSI-high or TMB-high cancers, may be eligible for treatment with pembrolizumab, a programmed death-1 (PD-1) inhibitor, given the its tissue agnostic approval for MSI-high cancers and the demonstrated responses of TMB-high solid tumors (96, 97).

Anaplastic Thyroid Cancer (ATC), with a median overall survival of 4 months, is considered one of the most aggressive and lethal malignancies and typically presents at a median age of 65-70 years (95–97). This most-aggressive thyroid cancer, with a 6-month OS of 35%, and disease-specific mortality approaching 100% is responsible for over half of the annual thyroid cancer-related deaths despite comprising only 1.5% of all thyroid cancers (98–100). These outcomes are despite aggressive multimodality treatment regimens including surgery (when feasible), traditional cytotoxic chemotherapy and radiation therapy. ATC is postulated to have the potential to arise either *de novo* or from pre-existing DTC. The coexistence of *BRAF*-mutated ATC with PTC described in several studies, suggests the potential of a common DTC origin for most of these tumors (101, 102). ATC has a higher relative tumor mutational burden (TMB) than DTC although overall the TMB for ATC is still lower than many other solid malignancies (100). The mutational profile of ATC tends to include accumulation of variations in tumor suppressor genes such as *TP53 and PTEN*; oncogenes such as *TERT* promoter, *RAS*, *BRAF*, and *PIK3CA*; oncogene-fusions such as *NTRK*, *RET*, and *ALK*; or through mismatch repairs (103). Given the aggressive nature of ATC, most often with surgically unresectable disease at presentation, and resistance to radioactive iodine, chemotherapies, and radiation therapy, all patients with suspected ATC are recommended to undergo expeditious histological confirmation, staging, and molecular testing and if a targetable mutation is identified, treatment should include directed therapies against this actionable target.

The most significant shift in the management of ATC to occur in decades was the afore mentioned combinatorial use of *BRAF/MEK* inhibitors (dabrafenib/trametinib) in ATC patients harboring a *BRAF*V600E mutation (83). Due to the potential for long turn-around times for traditional NGS testing, some centers employ a rapid PCR assay to detect *BRAF*V600E in DNA isolated from paraffin blocks (48–72-hour turnaround) or use peripheral blood NGS (cell-free DNA) which has sensitivity of 75%–90% and turnaround time of 3–7 days. These options may enable slightly earlier initiation of targeted therapies if they exist (104, 105). Mutation-specific immunohistochemistry for *BRAF*V600E can also be useful in expeditiously identifying patients who might benefit from approved targeted therapy, but requires substantial tissue *via* core needle biopsy, FNA cell block, or even surgical specimen due to the potential for false positives (106). When successful, *BRAF*-directed therapy can induce rapid and substantial disease regression and may eventually render previously inoperable disease amenable for surgical resection (107). For these patients with advanced stage ATC who are able to undergo complete locoregional surgical resection, one study has shown some of the highest survival rates ever reported for this disease with a 94% 1-year survival and an unmet median OS in a cohort of 20 patients (8 of 20 having stage IVC disease) having received BRAF-directed therapy followed by surgery (98).

Medullary Thyroid Cancer (MTC) arises from parafollicular C cells which are neuroendocrine in origin and accounts for about 2% of thyroid cancers. Although rare, MTC accounts for about 14% of annual deaths from thyroid cancer (108–110). MTC most often occurs sporadically (80%) with hereditary forms (20%) being associated with the multiple endocrine neoplasia (MEN) type 2 syndromes. These inherited forms of MTC are associated with genomic alterations of the *RET* proto-oncogene and are inherited in an autosomal dominant fashion. Patients diagnosed with MTC, regardless of disease stage, personal history of other endocrinologic disorder, or family history, should have genetic counseling and be tested for germline *RET* mutations (91). About 6% of MTC patients with no family history or other endocrinologic disorder to suggest MEN, are found to harbor a germline *RET* mutation prompting counseling and testing of family members. Somatic *RET* mutations are also found in approximately 50% of patients with sporadic MTC. Somatic mutations in *HRAS* (~25%), *KRAS*, and rarely *NRAS* genes, which are canonically mutually exclusive with *RET* mutations, have also been identified in sporadic MTC (111). About 20% of sporadic MTC harbor neither *RET* nor *RAS* gene alterations (112). Patients with advanced sporadic MTC should be offered molecular testing since somatic *RET* mutations have been shown to lead to more aggressive disease, including higher T- and N-stage, and increase the rate of distant metastasis (84, 108).

Currently, two MKIs, vandetanib and cabozantinib, are approved by the U.S. FDA for the systemic treatment of MTC and show improvement in progression-free survival (78, 79), both MKIs have a narrow therapeutic window and off-target kinase inhibition causes significant toxicities. Additionally, MTC can acquire gatekeeper resistance mutations at RET codon V808 rendering these therapies ineffective (91). Recently however, selective *RET* inhibitors have shown both promising efficacy and more favorable toxicity profiles (85). Selpercatinib (LOXO-292) is a selective *RET* kinase inhibitor potently effective against *RET* alterations, including gene fusions, oncogenic mutations, and even the V804 gatekeeper mutation. Early data from LIBRETTO-001, the phase I/II study of selpercatinib, showed 56% of patients with *RET*-mutant MTC previously treated with vandetanib and/or cabozantinib achieved objective responses with mostly grade 1 or 2 adverse events, prompting early approval by the FDA (113). Currently, an ongoing randomized trial is evaluating treatment-naїve patients with *RET*-mutant MTC, comparing selpercatinib with standard MKI therapy. Pralsetinib (BLU-667), another selective *RET* inhibitor, has been recently approved by the FDA for the treatment of patients with advanced or metastatic *RET*-mutant MTC (IC50 0.3–5 nM). This approval was based on early data from the phase I/II trial (ARROW) of pralsetinib showing a 65% objective response rate in patients with *RET*- mutant tumors, including patients with MKI resistant tumors and with known gatekeeper mutations (84). In this study, pralsetinib has been well tolerated with most treatment related adverse events being low grade and reversible (114).

In summary, the use of molecular testing in the identification of therapeutic targets can have significant clinical impact. We are undoubtedly only seeing the beginning of this new frontier. Knowledge of molecular mutations, fusions, and gene expression profiles, especially for the most advanced and aggressive forms of thyroid cancer will likely continue to drive drug discovery and development world-wide.

## Summary

Molecular testing of thyroid nodules and thyroid cancer has improved the diagnostic accuracy of indeterminate thyroid nodules and provides actionable information regarding tumor prognosis. Additionally, identifiable molecular variants and fusions inform clinicians of a patient’s eligibility for targeted systemic therapies in the important subset of thyroid cancer patients with metastatic, progressive, radio-iodine refractory disease. Future research should focus on the clinical utility of molecular information to change the clinical approach to patients with thyroid nodules. For example, prospective studies on the extent of surgery and the assessment of changes in factors such as tumor recurrence. Additionally, novel analyses to predict tumor behavior are warranted. Finally, the investigation of targeted therapies in the neo-adjuvant setting for thyroid cancer that presents aggressively is ongoing and may improve overall outcomes, for example, with improved opportunities for acceptable surgical outcomes in previously unresectable tumors.

## Author contributions

JP, JK, and EC wrote equal portions of the first draft of this review. All authors contributed to the article and approved the submitted version.
